# Healthcare-associated infections in pediatric cancer patients: results of a prospective surveillance study from university hospitals in Germany and Switzerland

**DOI:** 10.1186/1471-2334-8-70

**Published:** 2008-05-23

**Authors:** Arne Simon, Roland A Ammann, Udo Bode, Gudrun Fleischhack, Hans-Martin Wenchel, Dorothee Schwamborn, Chara Gravou, Paul-Gerhardt Schlegel, Stefan Rutkowski, Claudia Dannenberg, Dieter Körholz, Hans Jürgen Laws, Michael H Kramer

**Affiliations:** 1 Pediatric Hematology and Oncology, University Children's Hospital, Bonn, Germany; 2 Pediatric Hematology and Oncology, Department of Pediatrics, University of Bern, Bern, Switzerland; 3 Pediatric Hematology and Oncology, University Children's Hospital, Cologne, Germany; 4 Pediatric Hematology and Oncology, University Children's Hospital, Erlangen, Germany; 5 Pediatric Hematology and Oncology, University Children's Hospital, Würzburg, Germany; 6 Pediatric Hematology and Oncology, University Children's Hospital, Leipzig, Germany; 7 Pediatric Hematology and Oncology, University Children's Hospital, Düsseldorf, Germany; 8 Institute for Hygiene and Public Health, University of Bonn, Germany

## Abstract

**Background:**

Pediatric cancer patients face an increased risk of healthcare-associated infection (HAI). To date, no prospective multicenter studies have been published on this topic.

**Methods:**

Prospective multicenter surveillance for HAI and nosocomial fever of unknown origin (nFUO) with specific case definitions and standardized surveillance methods.

**Results:**

7 pediatric oncology centers (university facilities) participated from April 01, 2001 to August 31, 2005. During 54,824 days of inpatient surveillance, 727 HAIs and nFUOs were registered in 411 patients. Of these, 263 (36%) were HAIs in 181 patients, for an incidence density (ID) (number of events per 1,000 inpatient days) of 4.8 (95% CI 4.2 to 5.4; range 2.4 to 11.7; P < 0.001), and 464 (64%) were nFUO in 230 patients. Neutropenia at diagnosis correlated significantly with clinical severity of HAI. Of the 263 HAIs, 153 (58%) were bloodstream infections (BSI). Of the 138 laboratory-confirmed BSIs, 123 (89%) were associated with use of a long-term central venous catheter (CVAD), resulting in an overall ID of 2.8 per 1,000 utilization days (95% CI 2.3 to 3.3). The ID was significantly lower in Port-type than in Hickman-type CVADs. The death of 8 children was related to HAI, including six cases of aspergillosis. The attributable mortality was 3.0% without a significant association to neutropenia at time of NI diagnosis.

**Conclusion:**

Our study confirmed that pediatric cancer patients are at an increased risk for specific HAIs. The prospective surveillance of HAI and comparison with cumulative multicenter results are indispensable for targeted prevention of these adverse events of anticancer treatment.

## Background

Pediatric cancer patients have an increased risk of potentially life-threatening infectious [1-4] complications due to their underlying illnesses and intensive anticancer treatment [5-7]. Most of these infections are bloodstream infections (BSIs), most often associated with a central venous access device (CVAD) [8]. Healthcare-associated infections (HAIs) in pediatric cancer patients have been investigated in only a few single-center studies [9-12]. One additional European study included two pediatric oncology units [13] in a 6-month prospective survey using periodic chart review during hospitalization, but did not use adjusted definitions for immunocompromised pediatric patients [14].

The exact proportion of HAIs in these high-risk patients is unknown; two single-center prospective studies in pediatric hematology-oncology patients, excluding allogenic bone marrow transplantations, found an incidence of 20% [9] (ID 10.8/1,000 inpatient days) and 24% [10] (ID 17.7/1,000 inpatient days), respectively. One recent single-center study found an ID of 38.9 HAIs per 1,000 inpatient days in a pediatric stem-cell and bone-marrow transplantation unit [12], but the corresponding protocol referred only to patients with neutropenia [15].

In 1998, our group developed a module for the prospective unit-based surveillance of HAI in pediatric cancer patients: Onkopaed NKI. The results of a prospective single-center 10-month pilot study confirmed the feasibility of this module as well as the high risk of HAI and CVAD-associated BSIs in pediatric cancer patients [9]. The Onkopaed NKI module has already been used for prospective intervention studies on CVAD-associated infections [16], the restricted use of glycopeptides as a component of empirical antimicrobial therapy [17], and antifungal prophylaxis with itraconazole [18]. We now present the results of the multicenter prospective surveillance study for HAI and nosocomial fever of unknown origin (nFUO) in 7 German pediatric oncology centers from 2001 through 2005.

## Methods

The Onkopaed NKI module [9] uses standard methods of the Centers for Disease Control and Prevention (CDC; Atlanta, GA) [19] for the prospective unit-based surveillance of selected HAIs and nFUOs (occurring at least 24 hours after patients' admission) in pediatric cancer patients. The most important outcome parameter, the ID of events per 1,000 inpatient days, was calculated as the number of events divided by all inpatient days times 1,000. The ID of events per 1,000 inpatient device utilization days and the device utilization rate itself were calculated correspondingly. 'Multidrug-resistant isolates' referred to bacterial pathogens in vitro resistant to at least two of the corresponding first-line antimicrobials (ceftazidime, ceftriaxone, aminoglycosides, piperacillin-tazobactam). The surveillance module did not ask for admissions or numbers of individual patients in the participating institution during the surveillance period.

### Inclusion Criteria

All inpatients treated for at least 24 hours on the participating pediatric oncology ward were consecutively included in the unit-based surveillance study in terms of inpatient days, device days, and transplantation days. Data from patients who experienced at least one event (HAI or nFUO) were entered into the database. The age of the patient, malignancy and relapse status (first illness *vs *relapse) were documented as well as the type of CVAD, the presence of severe neutropenia at the time of event, clinical symptoms, and certain outcome parameters related to the type of HAI. The number of admissions to the unit was not documented in this study.

### Definitions

Fever was defined as body temperature > 38°C for more than 4 hours or once > 38.5°C. Severe neutropenia was defined as an absolute neutrophil count < 0.5 × 10^9^/L or as a decreasing leukocyte count < 1.0 × 10^9^/L without a differential count available.

Acute transplantation days were days with severe neutropenia following high dose chemotherapy as a conditioning regimen for autologous or allogenic stem-cell or bone-marrow transplantation (SCT, BMT).

The CDC's system of definitions for HAI was adjusted to the particular situation of this risk group of immunocompromized patients [14] (the complete definition set is available electronically at [20]).

The reference values for the assessment of vital signs were adjusted to the patient's age [21]. For all events, the two-step validation concept of the Onkopaed NKI software necessitated a consensus between the primary data management personnel (study nurse and infection control personnel) and one of the attending pediatric oncologists. Attributes of BSI were severity in terms of bacteremia, sepsis, septic shock and septic shock with multiorgan failure [21] and association with CVAD [8]. The term 'catheter-associated bloodstream infection' referred to a BSI in a patient with a central venous catheter in place without any additional clinically or microbiologically confirmed focus of infection. The EORTC consensus criteria were used for the definition of invasive aspergillosis; only 'probable' or 'proven' cases were included in the analysis [22].

*Clostridium difficile *associated disease (CDAD) was defined as symptoms of an abdominal infection starting at least 48 hours after hospital admission, and with a positive *C. difficile*-toxin cell culture assay from at least one stool specimen, leading to initiation of antimicrobial treatment directed against *C. difficile *for at least 5 days.

Mortality attributable to HAI was defined as the proportion of patients in whom the attending physicians stated that the HAI had contributed to the fatal outcome.

### Ethic Approval and Informed Consent

The study protocol was approved by the Ethics Committee of the Medical Faculty, University of Bonn, Germany. The patient or his/her legal guardians signed informed consent for participation before any data were included in the study. No patient or legal guardian refused participation.

### Statistical Analyses

Poisson estimates and their exact 95% confidence intervals (95%CIs) were calculated. The exact homogeneity of Poisson rates test, the exact Fisher-Freeman Halton test, Fisher's exact test, and exact Poisson regression were used where appropriate (with inpatient days as rate multiplier, performed to asses the evolution over time of HAI incidence). Two-sided tests were applied throughout, and *P*-values below 0.05 were considered significant. StatXact 6 and LogXact 6 software (both from Cytel Software Corp., Cambridge, MA, USA) were used for all analyses.

## Results

### Basic Data including Device Utilization

Seven pediatric oncology centers, all located at tertiary care university facilities, and denoted as C1 to C7, participated in this study for at least 6 consecutive months from April 01, 2001 to August 31, 2005. While C3 was a specialized unit for allogenic and autologous SCT and BMT, all other units offered conventional chemotherapy and radiotherapy, as well as high dose chemotherapy and autologous SCT to their patients. The study covered a total of 204 months of prospective surveillance in the 7 centers. They took part for 14, 53, 30, 6, 32, 41 and 28 months (C1 to C7). In total, data on 54,824 inpatient days were collected. The respective centers contributed 4%, 35%, 4%, 4%, 19%, 21% and 12% (C1 to C7) to these inpatient days (Table 1 shows the number and proportion of acute transplantation days).

**Table 1:** Number of surveillance days by risk category

Category	Days
Total inpatient days	54,824
Stem cell transplantation (autologous)#	1,041
Stem cell transplantation (allogenic)#	751
Total CVAD days	44,094
- Non-tunneled CVAD	1,143
- Port-type	15,600
- Broviac/Hickman	27,351
External CSF drainage days	187
Urinary catheter days	1,045

CVAD = central venous access device; CSF = cerebrospinal fluid (ventricle)

# Days with a total granulocyte count < 0.5 × 10^9^/L

**Figure 1:**
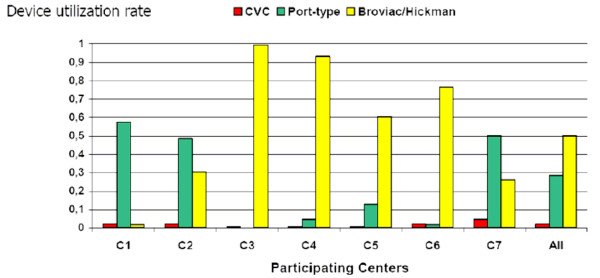
**Device utilization rates (inpatient utilization days/inpatient days) for the different central venous access devices (CVADs) in the participating centers (C1-C7)**. CVC = conventional non-tunneled central venous access. Port = Port type CVAD with subcutaneously implanted reservoir. Broviac/Hickman = tunneled long term CVAD with subcutaneous cuff.

The 7 centers differed significantly in the utilization rates of any CVAD (mean cumulative rate × 100: 80; range 62 for C1 to 100 for C3; *P *< 0.001; Figure 1), of urinary catheters (0 for C3 to 5.5 for C1; *P *< 0.001), and of CSF drainages (0 for C3/4/6 to 1.1 for C5; *P *< 0.001). As shown in Fig. 1, local preferences led to significantly different CVAD-specific utilization rates (*P *< 0.001). Further basic data, in particular those of cumulative device utilization days, are given in Table 1.

### Infections: Documented HAIs vs nFUOs

Of 727 events, 263 (36%; 181 patients) were HAIs and 464 (64%; 230 patients) were nFUOs. Table 2 lists patient characteristics related to HAI *vs *nFUO events.

**Table 2:** Basic characteristics of healthcare-associated infections (HAIs) and of nosocomial fevers of unknown origin (nFUO)

**Item**	**HAI (N = 263)**	**nFUO (N = 464)**	***P*-value**
Number of patients included (in total, n = 411)	181	230	-
Age at diagnosis of HAI/nFUO			< 0.001
< 5 years	84 (32%)	174 (38%)	
5 years to < 10 years	58 (22%)	130 (28%)	
10 years to < 15 years	56 (21%)	99 (21%)	
≥ 15 years	65 (25%)	61 (13%)	
Underlying disease			0.07
Acute lymphoblastic leukemia	86 (33%)	136 (29%)	
Acute myeloid leukemia	34 (13%)	40 (9%)	
Lymphoma	29 (11%)	48 (10%)	
solid tumor outside central nervous system	70 (27%)	166 (36%)	
solid tumor of central nervous system	34 (13%)	65 (14%)	
non-malignant hematologic disease	10 (4%)	9 (2%)	
Relapse of malignancy	54 (21%)	68 (15%)	0.049
Therapy^§ ^preceding HAI/nFUO			0.18
conventional chemotherapy	214 (81%)	391 (84%)	
high-dose chemotherapy & autologous SCT	22 (8%)	44 (9%)	
high-dose chemotherapy & allogeneic SCT	26 (10%)	25 (5%)	
radiation therapy	14 (5%)	22 (5%)	
Neutropenia at diagnosis of HAI/nFUO	145 (55%)	232 (50%)	0.19

Indicated are numbers (proportion of respective events).

^§^Since more than one treatment modality was applicable, the numbers do not add up to 263 and 464, respectively.

The age of patients with HAIs and nFUOs differed significantly, with a relative excess of HAIs in patients at least 15 years old. Besides, there was a significant relative excess of HAI in patients with relapsed malignancies. Neither malignancy diagnosis nor treatment modality preceding the event differed significantly between HAIs and nFUOs.

**Figure 2:**
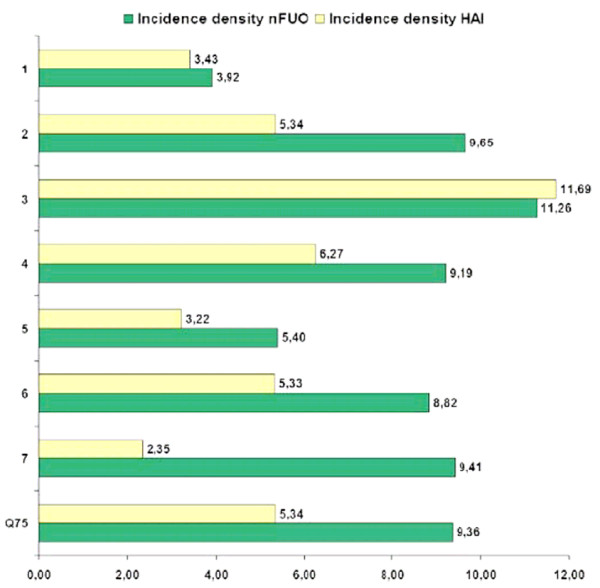
**Incidence density of 263 healthcare associated infections and 464 nosocomial fevers of unknown origin in the participating centers (C1-C7)**. Q75 = 75. percentile excluding data from center 3 (allogenic transplantation unit).

The 727 events recorded during 54,824 inpatient days resulted in an estimated overall ID of 13.3 events per 1,000 inpatient days (95% CI 12.3 to 14.3). The event IDs differed significantly among the 7 centers (range 7.4 for C1 to 23.0 for C3; *P *< 0.001), as did the ID of HAI (overall estimate 4.8; 95% CI 4.2 to 5.4; range 2.4 for C7 to 11.7 for C3; *P *< 0.001), and of nFUOs (8.5; 7.7 to 9.3; 3.9 for C1 to 11.3 for C3; *P *= 0.001; Figure 2). Except for C3, where HAI accounted for 51% of all documented events, the other centers concordantly documented more nFUOs than HAIs. C3 reported 27 HAIs in 30 months of prospective surveillance, with 3 HAIs during 91 days with severe neutropenia after autologous SCT and 24 HAIs during 751 days in severe neutropenia following allogenic SCT or BMT, for a local HAI ID of 33 after autologous, and of 32 after, allogenic transplantation. In the remaining centers, the ID of nosocomial infections/1,000 acute autologous transplantation days with neutropenia ranged from 0.0 (C7; 180 days, 0 HAI) to 47.4 (C2; 211 days, 10 HAI).

### Presence of Severe Neutropenia at Diagnosis of the HAI

The proportion of patients with severe neutropenia at the time of the event did not differ between HAIs and nFUOs (Table 2). All patients with blood-culture positive septic shock (N = 5) and blood-culture positive sepsis with multiorgan failure (N = 3) had presented with severe neutropenia, as did 83% of those with blood-culture positive sepsis (29 of 35), 80% with clinically documented sepsis (8 of 10, blood culture negative), 73% with invasive aspergillosis (19 of 26), and 52% with bacteremia (49 of 95). In patients with surgical site infections, pneumonia or *Clostridium difficile*-associated disease, the proportion of patients with neutropenia at the onset of the infection was 27%, 55% and 29%, respectively.

### Healthcare-associated infections: Etiology and Resistances

Of the 263 HAIs, 153 (58%) were BSIs (see Table 3 for details). Of the 138 blood-culture positive BSIs, 123 (89%) were associated with a CVAD, resulting in an overall ID of 2.8 per 1,000 utilization days (95% CI 2.3 to 3.3). The IDs were significantly different for the 3 types of CVADs: The ID was 5.2 in non-tunneled, short-term CVADs (95% CI 1.9 to 11.4; 6 events in 1,143 days), 1.8 in Port-type CVADs (1.2 to 2.6; 28 events in 15,600 days), and 3.3 in Broviac/Hickman catheters (2.6 to 4.0; 89 events in 27,351 days; *P *= 0.009). Only 15 (11%) of all BSI were secondary cases related to an identified source other than the CVAD. This corresponds to an ID of 0.27 secondary BSIs per 1,000 inpatient days. In 15 (13%) of 114 blood culture positive, CVAD-associated BSIs, the device was removed because of the infection (Port-type 7 of 28; Broviac/Hickman 8 of 86).

**Table 3:** Distribution and incidence densities of 263 HAI

**Kind of healthcare-associated infection (HAI)**	**No. (Proportion)**	**ID**
All HAIs	263 (100%)	4.80
**Bloodstream infections (BSI)**	**153 (58%)**	**2.79**
Laboratory-confirmed (blood-culture positive) BSI	138 (52%)	2.52
*Bacteremia*	*95 (36%)*	*1.73*
*Sepsis*	*35 (13%)*	*0.64*
*Septic shock*	*5 (2%)*	*0.09*
*Septic shock with multiorgan failure*	*3 (1%)*	*0.05*
Blood-culture negative BSI	15 (6%)	0.27
*Clinically defined sepsis*	*10 (4%)*	*0.18*
*Clinically defined septic shock*	*1 (0%)*	*0.02*
*Clinically defined septic shock with multiorgan failure*	*4 (2%)*	*0.07*
**Radiologically confirmed pneumonia**	**20 (8%)**	**0.36**
**Invasive aspergillosis**	**26 (10%)**	**0.47**
Pulmonary invasive aspergillosis	21 (8%)	0.38
*Proven pulmonary case of invasive aspergillosis*	*6 (2%)*	*0.11*
*Probable pulmonary case of invasive aspergillosis*	*15 (6%)*	*0.27*
Other localization (sinus, central nervous system)	5 (2%)	0.09
**Respiratory syncytial virus (RSV) infection**	**2 (1%)**	**0.04**
**Surgical site infection**	**15 (6%)**	**0.27**
Early (≤ 14 days from intervention) surgical site infection	6 (2%)	0.11
Late (> 14 days from intervention) surgical site infection	9 (3%)	0.16
***Clostridium difficile*associated enterocolitis**	**24 (9%)**	**0.44**
Preceding i.v. antibiotics within 14 days	11 (4%)	0.20
No preceding i.v. antibiotics within 14 days	13 (5%)	0.24
**Rotavirus associated enterocolitis**	**6 (2%)**	**0.11**
**Urinary tract infection (UTI)**	**8 (3%)**	**0.15**
UTI related to urinary tract catheter	3 (1%)	0.05
UTI not related to urinary tract catheter	5 (2%)	0.09
**Ventriculitis related to external CSF drainage**	**1 (0%)**	**0.02**
**Local infections at the central venous access device exit site**	**8 (3%)**	**0.15**

No. indicates the absolute number; ID indicates incidence density per 1,000 inpatient days

In the 138 blood-culture positive BSIs, 145 isolates out of 25 pathogenic species (144 bacteria and 1 *Candida albicans*) were detected. Seven BSIs (5%) were polymicrobial, and 62% of all isolates were Gram positive (Table 4). Coagulase-negative staphylococci (CoNS; 38% of them MRSE) were the most prevalent pathogens (34%). In addition to the 19 MRSE, only 4 (3%) of all isolates were multiresistant, with two vancomycin-resistant enterococci (VRE), one multiresistant *Klebsiella *spp. and one multiresistant *Pseudomonas aeruginosa*.

**Table 4:** Distribution of 145 pathogens in 138 laboratory-confirmed BSIs

**Isolate**	**No of Isolates (n = 145)**
CoNS (of these: methicillin-resistant)	50 (MRSE: 19)
*Escherichia coli*	22
α-hemolytic streptococci (Str. viridans)	21
*Klebsiella pneumoniae*	9
*Pseudomonas aeruginosa*	8
Staphylococcus aureus*	5
*Enterobacter cloacae*	4
*Enterococcus *spp.	3
*Acinetobacter woffii*	2
*Bacillus *spp.	2
*Micrococcus *spp.	2
*Neisseria *spp	2
*Pantoea agglomerans*	2
Vancomycin-resistant *Enterococcus faecium *(VRE)	2
*Arthrobacter *spp.	1
*Burkholderia cepacia*	1
*Strep. Pneumoniae*	1
*Corynebacterium *spp.	1
*Kocuria kristinae*	1
*Leuconostoc *spp.	1
MR-*Klebsiella *spp.	1
MR-*Pseudomonas *spp.	1
*Proteus *spp.	1
*Ralstonia pickettii*	1
*Candida albicans*	1

BSI = bloodstream infection

CoNS = coagulase-negative Staphylococci

MRSE = methicillin-resistant coagulase-negative Staphylococci

MR = multiresistant in vitro

(here: to piperacillin, aminogylcosides and to 3^rd ^generation cephalosporines)

* No MRSA was detected.

Twenty cases of radiologically confirmed nosocomial pneumonia were reported. None of these patients had been mechanically ventilated in the week before the event, and no additional risk factors were detected. In only one case of pneumonia could a causal pathogen be identified (*Acinetobacter baumanii*, detected in a bronchoalveolar lavage sample).

In the 26 reported cases of invasive aspergillosis, patients had been hospitalized for a median duration of 16 days (range 3 to 64). Only 2 respiratory syncytial virus (RSV) infections were reported from a single center (C2).

There were 16 pathogens detected related to the 15 surgical-site infections (SSIs), the most prevalent were *Staphylococcus aureus *(n = 7; 1 of these MRSA) and CoNS (n = 5; 2 of these MRSE). The other pathogens were *Escherichia coli, Enterobacter cloacae, Enterococcus faecium*, and *K. pneumoniae (one each)*. The median time from the operation to the diagnosis of SSI was 16 days (IQR 10–28 days). Five SSIs (30%) were related to a local infection of a central venous access device. In 7 of 15 SSIs (47%) treatment included second surgical intervention. One SSI (7%) was related to an orthopedic implant (osteosarcoma, femur, *S. aureus *infection).

In total, 24 cases of CDAD were reported. In 3 patients, surgical interventions were necessary during the clinical course of the acute intra-abdominal infection. In C6, 9 out of 13 cases were reported from June, 2001 to January, 2001, for an incidence density of 3.79 (9 events in 2,376 inpatient days). After the local preventive measures were reorganized, the ID decreased to 0.44 (4 cases in 9,077 days).

Six cases of rotavirus gastroenteritis were reported in patients with a median age of 2.9 years (range 1.2 to 12.6). Five of these required intravenous hydration and 2 experienced severe disturbances of electrolyte balance resulting in parenteral interventions. Of the 8 urinary tract infections reported, only 3 were catheter-related, for an ID of 2.9/1,000 device utilization days. A single case of a ventriculits/meningitis by CoNS related to external CSF drainage after CNS tumor surgery was reported, for an ID of 5.4/1,000 utilization days.

With 8 of 263 children's deaths attributed to an HAI, the overall attributable mortality was 3.0%. Severe neutropenia at time of HAI was not associated with a fatal outcome (3 of 145 patients with, *vs *5 of 118 without, severe neutropenia; *P *= 0.47). Six of these fatal outcomes were observed in patients with invasive aspergillosis (4 pulmonary and 2 cerebral), the other 2 fatal outcomes were in patients with blood-culture negative septic shock with multiorgan failure.

**Figure 3:**
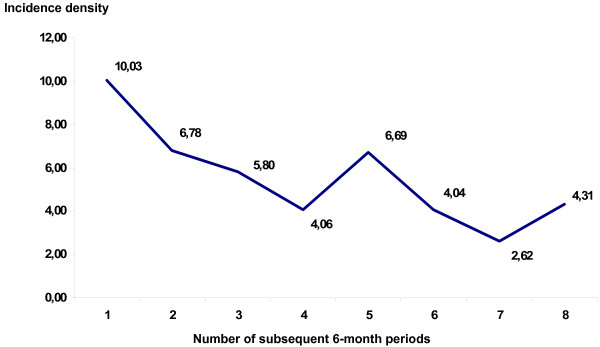
Incidence density of HAI over time in Center 2 (48 months of surveillance divided in 8 consecutive periods of 6 months).

Overall, there was a significant 16% reduction of HAI ID per year (odds ratio per year of study participation: 0.84; 95% exact CI 0.75 to 0.95; *P *= 0.005). Analyzing each center separately, there was a significant decrease in HAIs over time only in center 2 (0.75; 0.64 to 0.88; *P *< 0.001; Figure 3).

## Discussion

This study is the first prospective multicenter surveillance study of HAI in pediatric cancer patients using standardized CDC methods and adjusted consensus definitions for this special patient population. Our study confirms that pediatric cancer patients are at an increased risk for specific HAI. These adverse events can result not only in significant morbidity and mortality, but also in an increased expenditure of limited financial and personnel resources. In particular, CVAD-associated BSI, which represent the majority of all HAIs in this population, should be targeted to improve clinical practices and evaluate new preventive strategies [23].

Although associated with a severe clinical course, a significant proportion of all patients with HAI did not have neutropenia at the time of diagnosis. This is in agreement with results from other groups who found severe CVAD-related infections in pediatric cancer patients in the absence of neutropenia [24]. As a consequence, surveillance should not be limited to the period of neutropenia, and for the calculation of IDs days of neutropenia should not be used as the (sole) denominator.

Even in those patients who have received an allogenic BMT or SCT, the clinical relevance of certain late onset infections (BSIs or invasive fungal infections after the recovery of the leukocyte count have been observed) [7] argues against a surveillance that is restricted to early 'neutropenic' HAI.

The IDs described in our study are within the range of those from single-center studies. During 3 months of surveillance in 52 pediatric cancer patients with 135 admissions and 1,017 inpatient days, Urrea et al. [10] found a HAI rate of 23.5%, an incidence of 13.3/100 admissions and an ID of 17.7 per 1,000 patient days. In the pilot study of the Oncopaed NKI module [9], we investigated 40 HAIs in 28 out of 143 patients (HAI rate 19.6%) hospitalized for 3,701 days (776 admissions) during a 10-month prospective surveillance period. The HAI incidence was 5.2/100 admissions and the overall ID of HAI was 10.8 per 1,000 inpatient-days. The overall CVAD-related BSI rate was 7.4 per 1,000 inpatient utilization days.

In the transplantation unit (C3), patients after an autologous STC had the same ID for HAI as those after allogenic transplantation. This is in line with a prospective surveillance study in pediatric patients treated in an STC unit conducted by Laws et al. [12]. Using the surveillance protocol of the German National Reference Centre for Surveillance of HAI for adults [15], 34 pediatric and 39 adult patients were followed for 24 months. The incidence of HAI during neutropenia (1,156 neutropenic days) was 38.9 per 1,000 days, without significant difference between the groups. The Oncopaed NKI module may be used for the surveillance of late onset infections after SCT, in particular invasive aspergillosis [25].

Overall, the attributable mortality in our study was surprisingly low, mainly due to a low mortality in patients with BSIs. The outcome of BSIs in pediatric oncology patients has improved over the last decade, mainly due to early aggressive treatment in any suspected case of sepsis and due to advances in intensive care in this particular population [26].

The proportion of resistant isolates in the BSIs was also surprisingly low, considering the increasing proportion of resistant isolates in studies from other countries [13].

Wisplinghof et al. [27] determined antimicrobial susceptibilities of nosocomial BSI isolates in pediatric patients in the US SCOPE project. As in our study, Gram-positive pathogens accounted for most (65%) of the cases, Gram-negative pathogens for 24% and fungi for 11%. We identified only a single case of candidemia. In Wisplinghof's study, the proportion of MRSA increased from 10% in 1995 to 29% in 2001 [27]. Vancomycin-resistance was seen in 11% of *E. faecium *isolates (VRE). In our study, no BSI was caused by MRSA, but 2 of 5 *E. faecium *BSI were caused by VRE. In the study of Raymond et al. [13], the prevalence of antimicrobial resistance depended on the type of unit. Up to 26% of *S. aureus *and 89% of CoNS were methicillin-resistant (MRSA, MRSE), and 38% of *K. pneumoniae *expressed an extended-spectrum beta-lactamase (ESBL) [28].

The high mortality in patients with invasive aspergillosis, in particular in those patients with disseminated disease, is still a matter of great concern. The recognition of the role of external reservoirs and acute exposure to fungal spores as well as the expanding scope of the agents available for antifungal prophylaxis may contribute to a higher success rate in the prevention of these often fatal infections in high-risk pediatric cancer patients [18].

Based on our experience with a norovirus outbreak in pediatric oncology patients [29] and our recent investigation of rotavirus infections in a matched pairs analysis [30], the surveillance of gastrointestinal HAI in pediatric cancer patients should include at least rotavirus, norovirus, and CDAD, which is, however, difficult to define in this population [31].

Our results confirm the observation of Dettenkofer et al. [15] that a remarkable proportion of all symptomatic patients with CDAD do not suffer from neutropenia at the time of diagnosis. The empirical antimicrobial regimen of fever and neutropenia does not cover *C. difficile *and fosters the production of *C. difficile *toxins [32]. Discontinuation of the offending antibiotic as a general treatment principle in patients with CDAD [33] is not feasible in patients with neutropenia. Recently, hypervirulent nosocomially transmitted *C. difficile *isolates have been described in several countries, causing increased morbidity, length of hospital stay, and mortality in adult patients [34].

Considering the significant morbidity of affected patients, the possibility of outbreaks, and the emerging threat of hypervirulent isolates, it seems mandatory to implement continuous prospective surveillance of CDAD in pediatric cancer patients [35].

Prospective surveillance modules, such as that presented here, may be utilized to detect early (< 30 days) or late (up to 2 years in presence of prosthetic devices) SSI. Gaur et al. [36] retrospectively investigated infections complicating limb-sparing surgery (104 procedures) in 103 children and young adults with bone malignancies. They found a high incidence of infections (median 4%; range 0–13%), including local SSI in 67% and secondary bacteremia in 21% of patients. Patients who developed deep infections were more likely to undergo amputation (odds ratio [OR] 24.0; 95% CI 5.1–114.0; *P *< 0.001) and were less likely to have good functional outcomes (OR 0.02; 95%CI 0.002–0.15; *P *< 0.001). Different strategies of antimicrobial prophylaxis and antibacterial management in tumor-related orthopedic surgery may – as well as in other surgical interventions [37] – be evaluated prospectively in future studies [38].

The fact that we defined as nosocomially acquired infection only those RSV infections in which the first symptoms occurred after at least 5 days of inpatient care did not allow the inclusion of some cases of nosocomial RSV infection, since the exact onset is sometimes difficult to determine. Although RSV is easily communicable and has been the cause of fatal outbreaks even in the setting of allogenic BMT [39], there is still no evidence-based antiviral treatment option [40]. Therefore, the early detection of this pathogen and immediate contact/droplet isolation of infected patients and their relatives is of utmost importance.

Our results demonstrate that microbiologic testing in pediatric cancer patients with pneumonia, with the exception of mechanically ventilated patients with acute respiratory failure, often fails to identify the causative agent [6]. In the light of the profoundly restricted time budget of infection control personnel, the inclusion of pneumonias without a confirmed pathogen, blood-culture negative BSIs, urinary tract infections, and nFUOs should be reconsidered critically in surveillance efforts in this population.

While we did not control for confounding variables such as demographic characteristics of the patients, duration of neutropenia or illness severity, further studies are needed to confirm that participation in such a surveillance study results in a significant decrease in HAI rates. This was not an interventional study and each participating center decided on its own responsibility about any practical consequence related to the reported HAI rates.

Today, HAIs are considered to be important adverse outcomes in terms of increased morbidity and mortality, postponed chemotherapy treatment cycles, as well as prolonged duration of hospital stay and additional costs from the perspective of the caregiver. All efforts to prevent HAI and to contain the nosocomial spread of pathogens are critical aspects of patient's safety in the hospital environment [41]. Thus, the prospective surveillance of HAI and comparison with standardized rates should be recognized as a fundamental component of quality assurance and control in the treatment of immunocompromized pediatric cancer patients.

## Conclusion

Our multicenter study confirmed that pediatric cancer patients are at an increased risk for specific HAIs, in particular vascular catheter-associated bloodstream infections. It seems reasonable to implement a specific surveillance protocol for HAI in this high risk population. Surveillance efforts should not only focus on periods of profound neutropenia. The prospective surveillance of HAI and comparison with cumulative multicenter results are indispensable for targeted prevention of these adverse events of anticancer treatment.

## Competing interests

The authors declare that they have no competing interests.

## Authors' contributions

All of the authors contributed substantially to the study. AS was the principal investigator of this study and prepared the manuscript, RAA was the local investigator in Bern and performed the statistical analysis, UB and GF were additional investigators in Bonn, H–MW and DS were local investigators in Cologne, CG was the local investigator in Erlangen, P–GS and SR were local investigators in Wuerzburg, CD and DK were local investigators in Leipzig, HJL was the local investigator in Duesseldorf, MHK participated substantially in the development of the protocol, data analysis, and manuscript preparation. All authors read and approved the final manuscript.

## Pre-publication history

The pre-publication history for this paper can be accessed here:



## References

[B1] Haupt R, Romanengo M, Fears T, Viscoli C, Castagnola E (2001). Incidence of septicaemias and invasive mycoses in children undergoing treatment for solid tumours: a 12-year experience at a single Italian institution. Eur J Cancer.

[B2] Castagnola E, Cesaro S, Giacchino M, Livadiotti S, Tucci F, Zanazzo G, Caselli D, Caviglia I, Parodi S, Rondelli R, Cornelli PE, Mura R, Santoro N, Russo G, De Santis R, Buffardi S, Viscoli C, Haupt R, Rossi MR (2006). Fungal infections in children with cancer: a prospective, multicenter surveillance study. Pediatr Infect Dis J.

[B3] Castagnola E, Conte M, Parodi S, Papio F, Caviglia I, Haupt R (2007). Incidence of bacteremias and invasive mycoses in children with high risk neuroblastoma. Pediatr Blood Cancer.

[B4] Castagnola E, Molinari AC, Giacchino M, Chiapello N, Moroni C, Caviglia I, Fratino G, Haupt R (2005). Incidence of catheter-related infections within 30 days from insertion of Hickman-Broviac catheters. Pediatr Blood Cancer.

[B5] Gaur AH, Flynn PM, Shenep JL (2004). Optimum management of pediatric patients with fever and neutropenia. Indian J Pediatr.

[B6] Neville K, Renbarger J, Dreyer Z (2002). Pneumonia in the immunocompromised pediatric cancer patient. Semin Respir Infect.

[B7] Benjamin DK, Miller WC, Bayliff S, Martel L, Alexander KA, Martin PL (2002). Infections diagnosed in the first year after pediatric stem cell transplantation. Pediatr Infect Dis J.

[B8] Simon A, Bode U, Beutel K (2006). Diagnosis and treatment of catheter-related infections in paediatric oncology: an update. Clin Microbiol Infect.

[B9] Simon A, Fleischhack G, Hasan C, Bode U, Engelhart S, Kramer MH (2000). Surveillance for nosocomial and central line-related infections among pediatric hematology-oncology patients. Infect Control Hosp Epidemiol.

[B10] Urrea M, Rives S, Cruz O, Navarro A, Garcia JJ, Estella J (2004). Nosocomial infections among pediatric hematology/oncology patients: results of a prospective incidence study. Am J Infect Control.

[B11] Paulus SC, van Saene HK, Hemsworth S, Hughes J, Ng A, Pizer BL (2005). A prospective study of septicaemia on a paediatric oncology unit: a three-year experience at The Royal Liverpool Children's Hospital, Alder Hey, UK. Eur J Cancer.

[B12] Laws HJ, Kobbe G, Dilloo D, Dettenkofer M, Meisel R, Geisel R, Haas R, Gobel U, Schulze-Robbecke R (2005). Surveillance of nosocomial infections in paediatric recipients of bone marrow or peripheral blood stem cell transplantation during neutropenia, compared with adult recipients. J Hosp Infect.

[B13] Raymond J, Aujard Y (2000). Nosocomial infections in pediatric patients: a European, multicenter prospective study. European Study Group. Infect Control Hosp Epidemiol.

[B14] Carlisle PS, Gucalp R, Wiernik PH (1993). Nosocomial infections in neutropenic cancer patients. Infect Control Hosp Epidemiol.

[B15] Dettenkofer M, Ebner W, Bertz H, Babikir R, Finke J, Frank U, Ruden H, Daschner FD (2003). Surveillance of nosocomial infections in adult recipients of allogeneic and autologous bone marrow and peripheral blood stem-cell transplantation. Bone Marrow Transplant.

[B16] Simon A, Fleischhack G, Wiszniewsky G, Hasan C, Bode U, Kramer MH (2006). Influence of Prolonged Use of Intravenous Administration Sets in Paediatric Cancer Patients on CVAD-related Bloodstream Infection Rates and Hospital Resources. Infection.

[B17] Simon A, Groger N, Wilkesmann A, Hasan C, Wiszniewsky G, Engelhart S, Kramer MH, Bode U, Ammann RA, Fleischhack G (2006). Restricted use of glycopeptides in paediatric cancer patients with fever and neutropenia. Int J Antimicrob Agents.

[B18] Simon A, Besuden M, Vezmar S, Hasan C, Lampe D, Kreutzberg S, Glasmacher A, Bode U, Fleischhack G (2006). Itraconazole prophylaxis in pediatric cancer patients receiving conventional chemotherapy or autologous stem cell transplants. Support Care Cancer.

[B19] National Nosocomial Infections Surveillance (NNIS) System (2004). National Nosocomial Infections Surveillance (NNIS) System Report, data summary from January 1992 through June 2004, issued October 2004.. Am J Infect Control.

[B20] Simon A Oncoped Study website. http://www.onkopaednki.de/.

[B21] Goldstein B, Giroir B, Randolph A (2005). International pediatric sepsis consensus conference: definitions for sepsis and organ dysfunction in pediatrics. Pediatr Crit Care Med.

[B22] Ascioglu S, Rex JH, de Pauw B, Bennett JE, Bille J, Crokaert F, Denning DW, Donnelly JP, Edwards JE, Erjavec Z, Fiere D, Lortholary O, Maertens J, Meis JF, Patterson TF, Ritter J, Selleslag D, Shah PM, Stevens DA, Walsh TJ (2002). Defining opportunistic invasive fungal infections in immunocompromised patients with cancer and hematopoietic stem cell transplants: an international consensus. Clin Infect Dis.

[B23] O'Grady NP, Alexander M, Dellinger EP, Gerberding JL, Heard SO, Maki DG, Masur H, McCormick RD, Mermel LA, Pearson ML, Randolph A, Weinstein RA, Raad (2002). Guidelines for the prevention of intravascular catheter-related infections. Infect Control Hosp Epidemiol.

[B24] Aledo A, Heller G, Ren L, Gardner S, Dunkel I, McKay SW, Flombaum C, Brown AE (1998). Septicemia and septic shock in pediatric patients: 140 consecutive cases on a pediatric hematology-oncology service. J Pediatr Hematol Oncol.

[B25] Upton A, Kirby KA, Carpenter P, Boeckh M, Marr KA (2007). Invasive aspergillosis following hematopoietic cell transplantation: outcomes and prognostic factors associated with mortality. Clin Infect Dis.

[B26] Haut C (2005). Oncological emergencies in the pediatric intensive care unit. AACN Clin Issues.

[B27] Wisplinghoff H, Seifert H, Tallent SM, Bischoff T, Wenzel RP, Edmond MB (2003). Nosocomial bloodstream infections in pediatric patients in United States hospitals: epidemiology, clinical features and susceptibilities. Pediatr Infect Dis J.

[B28] Paterson DL, Bonomo RA (2005). Extended-spectrum beta-lactamases: a clinical update. Clin Microbiol Rev.

[B29] Simon A, Schildgen O, Maria Eis-Hubinger A, Hasan C, Bode U, Buderus S, Engelhart S, Fleischhack G (2006). Norovirus outbreak in a pediatric oncology unit. Scand J Gastroenterol.

[B30] Rayani A, Bode U, Habas E, Fleischhack G, Engelhart S, Exner M, Schildgen O, Bierbaum G, Maria Eis-Hubinger A, Simon A (2007). Rotavirus infections in paediatric oncology patients: A matched-pairs analysis. Scand J Gastroenterol.

[B31] van de Wetering MD, Kuijpers TW, Taminiau JA, ten Kate FJ, Caron HN (2003). Pseudomembranous and neutropenic enterocolitis in pediatric oncology patients. Support Care Cancer.

[B32] Donskey CJ (2006). Antibiotic regimens and intestinal colonization with antibiotic-resistant gram-negative bacilli. Clin Infect Dis.

[B33] Brook I (2005). Pseudomembranous colitis in children. J Gastroenterol Hepatol.

[B34] McDonald LC, Killgore GE, Thompson A, Owens RC, Kazakova SV, Sambol SP, Johnson S, Gerding DN (2005). An epidemic, toxin gene-variant strain of Clostridium difficile. N Engl J Med.

[B35] Bartlett JG, Perl TM (2005). The new Clostridium difficile--what does it mean?. N Engl J Med.

[B36] Gaur AH, Liu T, Knapp KM, Daw NC, Rao BN, Neel MD, Rodriguez-Galindo C, Brand D, Adderson EE (2005). Infections in children and young adults with bone malignancies undergoing limb-sparing surgery. Cancer.

[B37] Neville HL, Lally KP (2001). Pediatric Surgical Wound Infection.. Seminars in Pediatric Infectious Diseases.

[B38] Trampuz A, Zimmerli W (2006). Antimicrobial agents in orthopaedic surgery: Prophylaxis and treatment. Drugs.

[B39] McCann S, Byrne JL, Rovira M, Shaw P, Ribaud P, Sica S, Volin L, Olavarria E, Mackinnon S, Trabasso P, VanLint MT, Ljungman P, Ward K, Browne P, Gratwohl A, Widmer AF, Cordonnier C (2004). Outbreaks of infectious diseases in stem cell transplant units: a silent cause of death for patients and transplant programmes. Bone Marrow Transplant.

[B40] Hicks KL, Chemaly RF, Kontoyiannis DP (2003). Common community respiratory viruses in patients with cancer: more than just "common colds". Cancer.

[B41] Coffin SE, Zaoutis TE (2005). Infection control, hospital epidemiology, and patient safety. Infect Dis Clin North Am.

